# Managing Tenders in the Procurement of Advanced Medical Devices: An Original Model Based on the Net Monetary Benefit Combined With Three Clinical Endpoints

**DOI:** 10.7759/cureus.39062

**Published:** 2023-05-15

**Authors:** Andrea Messori, Sabrina Trippoli, Valeria Fadda, Maria Rita Romeo

**Affiliations:** 1 Health Technology Assessment (HTA) Unit, Regione Toscana, Firenze, ITA; 2 Pharmacology and Therapeutics, ESTAR, Firenze, ITA; 3 Biomedical Engineering, Fondazione Toscana Gabriele Monasterio, Pisa, ITA

**Keywords:** value-based purchasing, procurement, quality-adjusted life years, cost-effectiveness analysis, economic health evaluation

## Abstract

In medical devices, recent studies have proposed original approaches for standardizing competitive tenders with the aim of promoting reproducibility, avoiding discretional decisions, and applying value-based principles. In the framework of tenders' standardization, the net monetary benefit (NMB) method has attracted much interest, but its mathematical complexity has prevented a wide application. In the present work, we developed a procurement model that simplifies clinical information management for high-technology devices purchased for our public hospitals. Our objective was to promote the application of NMB in competitive tenders, particularly at the final stage of the procurement process, where the tender scores are determined. Software to facilitate this task in everyday practice has been developed. This software is made available through the present technical report. We surveyed the most relevant literature about NMB to select the main models commonly used in the studies published thus far. Standard equations of cost-effectiveness were identified. A simplified computation model based on three clinical endpoints was developed to estimate the NMB with less mathematical complexity. This model is proposed as an alternative to the standard approach based on a full economic analysis. The model developed herein has been implemented in a web-based software freely available on the Internet. This software is accompanied by a detailed description of the equations by which the NMB is estimated. A detailed application example is reported; a real tender carried out in 2021 has been re-examined for this purpose. In this re-analysis, the new software has been used to calculate the NMB of three devices. To our knowledge, this is the first experience in which an institution of the Italian healthcare system has evaluated the NMB as a tool for determining tender scores. The model is designed to offer performance similar to a full economic analysis. Our preliminary results are encouraging and suggest a wider application of this method. This approach has important implications regarding cost-effectiveness and cost containment because a value-based procurement is known to maximize effectiveness without determining an increase in costs.

## Introduction

The net monetary benefit (NMB) is a tool of cost-effectiveness analysis proposed to manage the procurement of medical devices according to value-based principles [1-19]. Its application has increased over the past 5 years, but more extensive use is advocated. Recent proposals have suggested a role for the NMB in the management of rankings of competitive tenders and in assessing the price of innovative devices. To our knowledge, no reports have described any systematic application of the NMB in the procurement of medical devices, although the potential advantages of this approach are widely recognized.

The current literature offers a few reports about the application of NMB in the field of high-technology medical devices, especially in competitive tenders. Owing to the mathematical complexity of the method, these types of advanced tenders have always been managed by developing de novo original software for each tender. This software aims to implement the complex computations required by the specific analysis and the specific clinical problem addressed by the tender. Of course, this approach cannot be proposed as a method to carry out all tenders regularly; for example, the need to enroll specialized personnel in the tenders organized this way [4] hampers a regular use of tenders in the procurement of high-technology devices; in contrast, a growing use of tenders in the management of advanced devices is frequently recommended as a means of promoting the application of cost-effectiveness to medical devices.

In the present work, we describe an original experience focused on using the NMB in procuring advanced medical devices. In particular, a general-purpose software (denoted in this report as "simplified software") is described that implements the model of NMB irrespective of the specific device and the specific clinical condition treated by the device. This tool aims to extend the use of NMB in tenders and separate the application of these approaches from the presence of specialized personnel. In more detail, the present project is based on a simplified methodological approach in which the mathematical complexity has been reduced by restricting the model to three clinical endpoints. Furthermore, the model is run using standard cost-effectiveness equations and, quite importantly, avoids using programming cycle techniques (such as those typically required by Markovian modeling). The three clinical endpoints can be incorporated de novo in the software whenever a new tender is started. Finally, the software has been made available on the Internet; its PHP/HTML code can be obtained from the authors upon request.

The examples of the application of NMB presented in this paper are based on previously published real-world data. In particular, a real tender carried out in 2021 [4] in an Italian region has been retrospectively re-assessed to test the performance of the simplified software; this tender deals with three stents employed in patients subjected to carotid endarterectomy.

## Technical report

The basic methodological features of the NMB are presented to explain how this parameter works throughout the tender process and, in more detail, how quality-adjusted life years (QALYs) are constructed as the product of life years (LYs) multiplied by a utility. On the other hand, costs are simply computed by multiplying the cost per event by the number of events. All calculations are normalized to the time horizon of the analysis. To summarize the theoretical basis of the method, the main methodological clue is that the NMB can be considered an estimate of the final tender score, which is the parameter that expresses the result of the procurement pathway. Regarding the computations performed by the simplified software, Table 1 narratively describes the subsequent computation phases that lead to the estimation of the NMB.

**Table 1:** In the three endpoint models, the endpoints selected to express the outcome of the stenting procedure include death, major stroke, and minor stroke; these three endpoints also appear in the output printed by the software. Abbreviations: QALDs, quality-adjusted life days; QALYs, quality-ajusted life years.

Step	Description	Numerical example
1.	Selecting the time horizon	12 months
2.	Estimating QALDs over time horizon (at baseline utility in the absence of events)	QALDs=339.45
3.	Estimating disutility in QALDs related to the endpoints	QALDs=26.2625
4.	Subtracting QALDs of disutility related to the endpoints	QALDs=313.1875, i.e., QALYs=0.8580
5.	QALDs converted into euro according to the WTP threshold	51,482.88 euro
6.	Estimating costs related to the endpoints	891.5365 euro
7.	Subtracting costs related to the endpoints; result=	50,591.3436 euro
8.	NMB	the above value

In handling the data of quality-adjusted survival, some obvious approximations are allowed; for example, a loss in the utility of 0.50 lasting one month has the same effect on quality-adjusted survival as a loss in the utility of 0.25 lasting two months. The general relationship is the following:

(adjusted disutility) = (real disutility) x (real duration) / (time horizon)

For example, in an analysis based on a time horizon of 12 months, an infection causing a loss in the utility of 0.30 lasting 1 month can be managed as a disutility of 0.025 lasting 12 months.

Study design

The objective of this section is to offer some detailed examples in which the application of the three-endpoint model is described analytically. In this framework, the performance of the "simplified software" has been compared with that of a full economic analysis with no restrictions on the number of endpoints; the full analysis was conducted in a tender run in Tuscany in 2021 [4]. This tender dealt with carotid stents and assessed three devices (Table 1). Complete clinical information is reported herein for each of these devices. In the "simplified" model, the three endpoints include death, major stroke, and minor stroke. Figure 2 focuses on the stent by Abbott, which won the real tender; this figure shows the event rates observed for the three endpoints, along with the cost implications associated with each endpoint. For the stent by Abbott, the rates of occurrence of the three endpoints (at 12 months) were the following: a) minor stroke, 2.35%; major stroke, 0.16%; death, 0.07%. For the stent by Cardinal Health, the rates were: a) minor stroke, 1.69%; major stroke, 1.04%; death, 0.93%. For the stent by Johnson & Johnson, the rates were a) minor stroke, 0.89%; major stroke, 3.12%; death, 1.78%. The price offers were: Abbott, 530 euros; Cardinal Health, 508 euros; Medtronic, 418 euros. The three clinical endpoints also appear in the final output generated by the software (Figure 2); this output also presents the values of utility and cost ("fixed parameters" of the tender) associated with each of these three endpoints. Regarding the other two devices of the lot (i.e., those manufactured by Cardinal Health and Medtronic; see Table 1), their examples are qualitatively identical in terms of computation pathway, but -of course- the numerical values differ from those of the device by Abbott.

As shown in Table 2, the values of NMB estimated by the three endpoint models were in complete qualitative agreement with the values originally estimated through the full economic analysis. A complete agreement between the two estimates was not expected owing to the influence of the adjustments resulting from reparametrizations; quite importantly, it should be stressed that theoretical considerations allow us to exclude these reparametrizations can modify the final rankings of the tender if the three-endpoint model replaces the full economic analysis.

**Table 2:** Retrospective analysis of a real tender carried out in 2021: characteristics of three devices included in the tender. The tender for the procurement of carotid stents is an example to test the three endpoint model: three stents were retrospectively assessed; the table compares the NMB estimated using the three-endpoint model with the NMB originally estimated in the real tender using a full economic analysis. NMB: net monetary benefit

Manufacturer	Price offered (euro)	NMB (euro) estimated by full model	NMB (euro) estimated by three-endpoint model
ABBOTT	530*	54,836*	50,591
CARDINAL HEALTH	518*	54,068*	41,118
MEDTRONIC	408*	52,854*	40,900

The issue of the influence of reparameterizations on the design of the computations required by the NMB is separately discussed in Appendix 1. It should be stressed that the NMB can be handled using a basic (and very simple) computation model in which a single scale is used that spans from 0 to infinity and retains the units of the currency (e.g., euro) in which the original data are expressed. Alternatively, to meet a very common administrative requirement recommending the use of two separate scales for the final results (i.e., a 0-30 scale for prices and a 0-70 scale for benefits), a more complex model can be used to implement the two scales mentioned above; in this second case, a complete reparameterization of all the data of the analysis is needed, and a more complex model must be used in which the final results are calculated according to the two separate scales. Briefly, the main difference between the simpler and more complex models is that, in the first case, all the data are kept in their original currency (the euro in the examples presented herein). In contrast, in the more complex model, a third monetary unit is needed to which the parameters employed in the two scales must be converted through reparameterizations. Finally, regarding reparameterizations, two-scale vs. one-scale designs, and VMU, these issues are separately discussed in Appendix 1; a detailed example based on this approach is not directly presented in the present article but can be found in reference [4].

**Figure 1:**
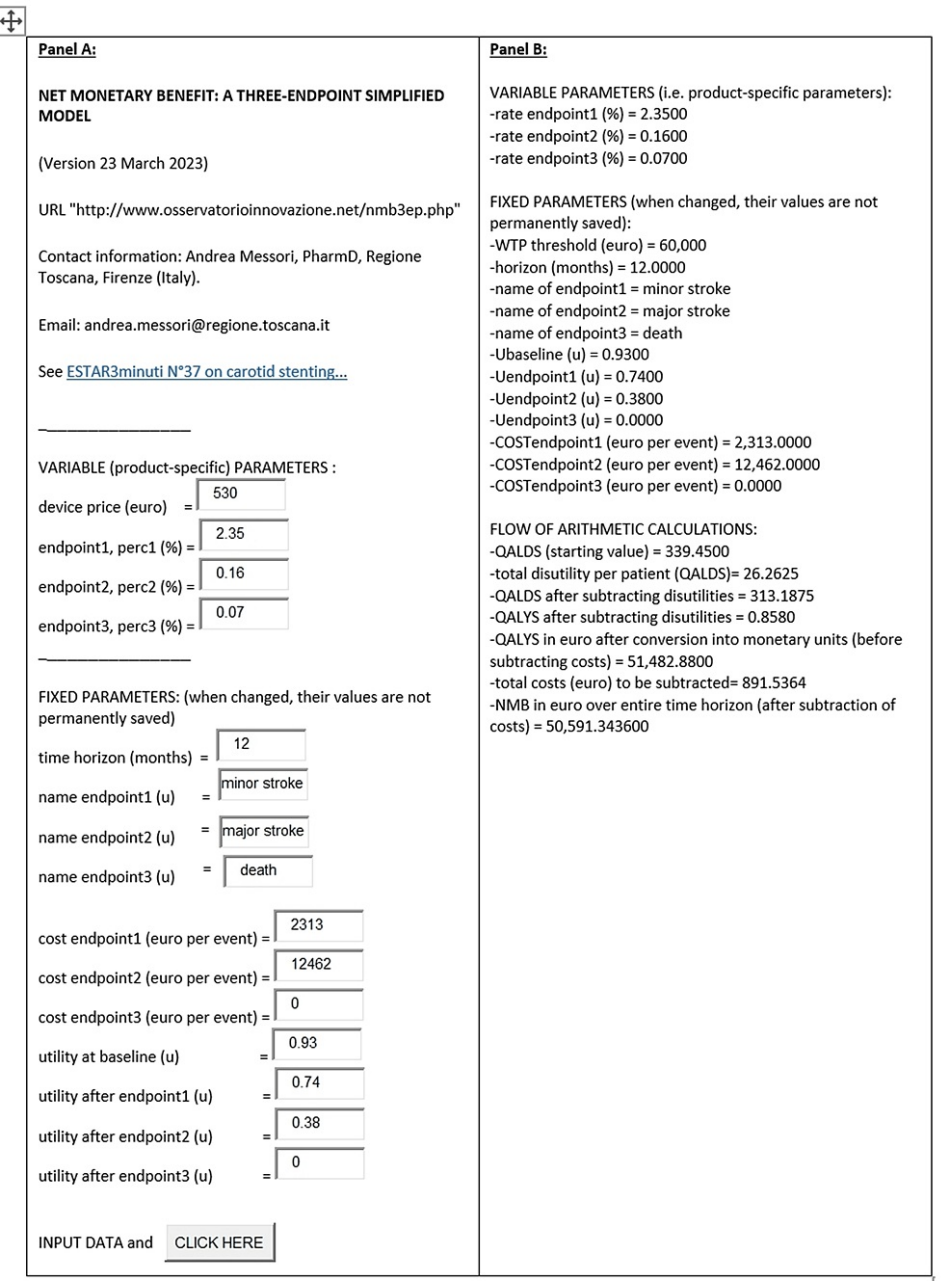
Software output Example of NMB estimation: output generated by the web-based software for the device by Abbott taken as an example (Panels A and B). Abbreviations: QALDS, quality-adjusted life days; QALYS, quality-adjusted life years.

It is worthwhile to recall the technical terms involved in the methodological process of NMB estimation. The main computation phases of the three-endpoint model have already been summarized in Table 1. In Figure 1, the lists are reported of the so-called "fixed parameters" (which are identical for all devices) along with the list of outcome parameters (which are specific for the specific device for which the tender score is being estimated).

Synopsis of the basic features of the NMB. This section reports the definitions of the main technical terms employed in the methodology of NMB [1-14]. In particular, the following five technical terms deserve to be specifically explained: a) NMB: this parameter, expressed in euro when managed in the European context, represents the final result of the application of the "simplified" method; b) technical college: this is the group of healthcare professionals who, at each tender, take the responsibility to write the technical specifications of the tender; in this phase, the three clinical endpoints are selected; c) willingness to pay (WTP) threshold: in the present work, reference has been made to the threshold of 60,000 euro per QALY gained, which is a quite common international standard; d) tender scores and ranking: regarding a tender that has evaluated N devices, the results -in the first place- include N ranking scores (the values of which are determined by application of the NMB); then, these N values are ranked in decreasing order, and this final classification represents the result of the tender; the device with the highest score wins the tender;

e) reparametrization of the quality scores: in the description of our examples, the NMB has generally been expressed on a single scale ranging from 0 to infinity. Reparameterizations are instead typical of tenders where two separate scales are employed. Because the tenders presented in this paper do not generally present any reparameterizations, and given that the reparametrization is not essential to the final result of the tender and, at the same time, makes the issue of computations much more complex, the topic of reparameterization has been extensively discussed only in the appendix (Appendix 1).

The flow of the main calculations performed by the software. In Figure 1, the same information reported in Table 1 is presented again in the form of the output generated by the web-based software.

## Discussion

In running a competitive tender regarding a group of similar devices, the NMB has a specific function in terms of cost-effectiveness because this parameter examines the main factors of cost and effectiveness in numerical terms and therefore guides the decision process toward the final ranking based on well-documented parameters. Given that the devices included in the tender are similar but not numerically identical regarding their action on the patients and given that also the prices offered differ from one another, the NMB also has the objective to explore the differences emerging between the different devices and to incorporate these differences into the final ranking. There is generally little recognition that, in the calculations determining the tender scores for each device, the NMB plays the role of "precursor" of the final classifications because the NMB exactly analyzes the same items (i.e., clinical effectiveness, economic consequences, and cost of the device) that participate in the tender scores. Expanding the awareness of this methodological property of the NMB is important because knowing this information is the prerequisite to promoting increased use of tenders in procuring high-technology devices and consequently improving the role of value-for-money in the acquisition of these products.

The main advantages of the NMB are the following: the NMB first evaluates clinical benefits as a favorable component and costs as a negative one and then balances their opposite effects in determining the final result for the device; a verification is constantly made that all prices offered for the devices are kept lower than the starting price of the bid; the QALYs represent the methodological tool through which increased tender scores are assigned to the devices showing more clinical effectiveness.

It should be stressed that, in this methodological framework, a single parameter (i.e., the NMB) allows us to perform the numerous functions required by the tenders' decision process to generate the final results. All in all, the principles of cost-effectiveness and value for money are put into practice, allowing us to maximize the amount of health generated for equal amounts of money spent. On the other hand, tenders designed according to the NMB possess a fundamental advantage in that their rankings directly correlate with the clinical and economic evidence available for each device; in this way, the results of the tender are made objective to a greater extent than those resulting from "traditional" tenders.

To examine this issue in more general terms, other two aspects deserve to be discussed when tenders are managed according to the model of NMB: firstly, benefits are quantified in monetary terms by combining the concept of quality-adjusted life years (QALYs) with that of WTP threshold; secondly, one should keep in mind that the condition needed to win the tender is that, by balancing costs and effectiveness, the device showing the best balance between these two components wins the tender, even though other devices have offered a lower price.

The NMB is a parameter that belongs in the same area as the ICER. These two parameters share some common features because they both require a pre-defined WTP threshold as well as the acceptance of QALYs as the parameter that expresses clinical benefits. Despite this common property, the differences between these two parameters are important and involve both theoretical and practical issues that can be summarized as follows: (i) the ICER is more suitable for speculative pharmacoeconomic research, whereas the NMB has a more "practical" nature and deals with the application of pharmacoeconomics to real life and to multiple comparisons as opposed to the binary comparison of two products typically managed by the ICER; (ii) from a mathematical viewpoint, the ICER is more complex than the NMB because the relationship between the ICER and costs is nonlinear while the relationship between NMB and costs is linear (or, more specifically, "additive" because items of benefit and items of cost are summed up algebraically with one another); (iii) as a matter of fact, the ICER is currently used much more frequently than the NMB, but this simply reflects the greater use of pharmacoeconomics for speculative purposes than for practical applications; (iv) the "typical" health-care professionals, who are likely involved in real-life pharmacoeconomic decisions, are more familiar with the ICER than with the NMB, whereas the opposite would be preferable; in fact, one advantage of the NMB is that three or more comparators can be managed whereas the typical analysis made through the ICER has a binary design.

**Figure 2:**
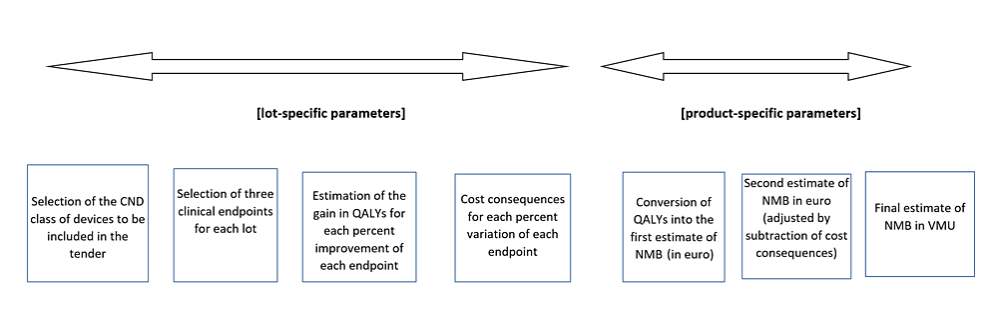
Typical phases through which a tender based on the NMB proceeds. Abbreviations: CND, Classificazione Nazionale dei Dispositivi; QALYs, quality-adjusted life years; NMB, net monetary benefit; VMU, virtual monetary units.

Regarding the areas where the ICER and/or the value-based price deserve to be applied, they especially include the case of innovative devices, in which innovation can be managed according to recent definitions [16]. Also, tenders are suitable for the application of value-based pricing [17]. In both instances, value-based prices can be useful either to guide the first purchase of a new device or to determine the starting price of a tender lot. In Figure 2, a flow chart is presented that describes how a typical tender based on the three-endpoint model can proceed. There is one phase in which, for the endpoint selected, the lot-specific parameters (or "fixed parameters") are identified and another phase in which product-specific outcome parameters are sought. If we consider our example of the outcomes observed in patients subjected to stent endarterectomy, the endpoints, such as minor/major strokes and death, along with their economic consequences, have been selected qualitatively as lot-specific outcome parameters. In a subsequent phase, each device has been associated with the rates observed for the endpoints selected ("variable" or device-specific parameters). This overall body of information is managed by the tender software, which eventually estimates the value of NMB for each device.

In conclusion, this article has presented a considerable amount of information (i.e., methods, clinical-economic data, and software) that facilitates the application of value-based procurement to tenders designed for advanced medical devices. In this context, the objective measurement of clinical benefit in a qualitative evaluation is crucial, but translating these methods from theory to practice encounters formidable obstacles, as demonstrated by the lack of examples published in the medical literature and on the Internet.

## Conclusions

In the area of in-hospital procurement of medical devices, the current degree of methodological advancement is very rudimental, and so the current developments are still aimed at an initial transition from no application at all of evidence-based medicine and cost-effectiveness practice to a very basic adoption of these methods. For example, an important task is represented by adopting the willingness to pay threshold, which is currently completely ignored by most Western hospitals' procurement offices. The landscape in the governance of high-tech devices shows a rapid evolution in the field of cost-effectiveness analysis and value-based pricing. The current scenario is strongly influenced by the lack of a European regulatory agency and, simultaneously, by the clearly insufficient economic governance managed by institutions, especially for high-tech devices. Compared to drugs, the difference is remarkable and, above all, appears to be further accentuating. Since there is no prospect of establishing a national (or European) organization aimed at the governance of devices, the most probable location of new activities regarding the economic governance of devices (including prices) is at the regional level. In Italy, this picture is gradually consolidating, and in this framework, tenders emerge as the most effective tool to put the principles of health-technology assessment (HTA) into our everyday practice.

The methodological advancements in this field focus on the clinical benefit as the main driver of the modern procurement process and attribute an increasing role to the principles of HTA (e.g., recognition of a WTP threshold, the role of clinical evidence, increased reproducibility of the decision process of tenders, adoption of more objective decision criteria, etc). In procuring innovative devices, the methodological tools described in the present article are suitable to promote an increased application of value-based pricing. Finally, regarding the devices purchased through tenders, the software described herein can have a fundamental role in recognizing the clinical benefit as the basic criterion of the decision process.

## Appendices

Data availability

A simple computational tool incorporating this value-based method is available at the following Internet address: https://www.multicentredatabase.net/nmb3ep.php. This software's PHP/HTML code can be obtained from the authors upon request; this code can be modified with no restrictions and can be uploaded, if needed, on a different website. We confirm that we developed the software ourselves, and it should be pointed out that the mathematical complexity of the method is minimal. The main programming difficulty lies in the PHP/HTML scripts that manage the queries with questions and answers that we have developed using general-purpose pre-programmed masks.

Appendix 1: Reparameterization of Tender Scores

This Appendix discusses the methods and the objectives of the reparameterization procedures that can be performed after the completion of a NMB analysis to split the final result of the NMB estimation into two separate values adapted, respectively, to the 0-30 scale for price and to the 0-70 scale for benefits. Table 3 summarises the main differences between the single-scale and the two-scale designs.  Regarding the model in which two scales are used  (0-30 and 0-70) and the VMU is the currency of all economic data, the basic design of this approach is explained in Table 3 and at point (d) in the Technical Report section; a detailed example of application is not presented in the article, but can  be found in reference 4.

**Table 3:** Panels A and B. Both Panels refer to the comparison between Device A and Device B performed according to the NMB approach; the six parameters involved in this hypothetical analysis are denoted as parameter A1, A2, A3, B1, B2, and B3. In Panel A, the analysis is simpler and is based on a single-scale design while  in Panel B, the analysis is more complex and is based on a two-scale design. Characteristics of the analysis in Panel A: 1) units of measurement: euro; 2) scale: from 0 to infinity; 3) multiplicative conversion factors: not employed; 4) design of computations: purely additive. Characteristics of the analysis in Panel B: 1) units of measurement: virtual monetary units; 2) scale: from 0 to 30 for price and from 0 to 70 for all the remaining items; 3) conversion factors: yes [purely additive when indicated by the symbol (*) or multiplicative when indicated by the symbol (**)]; 5) design of computations: multiplicative or additive. Parameters denoted with the (*) symbol generate the corresponding parameters through additions or subtractions without using any multiplication (according to the algebraically additive -or “net”- computations typical of the NMB); parameters denoted with the (**) symbol generate the corresponding parameters through multiplicative conversion factors. § Expressed in euro §§ Expressed in virtual monetary units (VMU).

	Panel A: Single-scale design						
	Device A			Device B			
Qualitative analysis	Clinical items (converted into monetary units): parameter A1 (*)	All costs (except device price): parameter A2 (*)	Device price parameter A3 (*)	Clinical items (converted into monetary units): parameter B1 (*)	All costs (except device price): parameter B2 (*)		Device price parameter B3 (*)
	-positive impact	-negative impact	-negative impact	-positive impact	-negative impact		-negative impact
From qualitative to quantitative analysis	overall result for device A (from A1, A2, and A3)			overall result for device B (from A1, A2, and A3)			
Quantitative analysis	Clinical items (converted into monetary units)	All costs (except device price)	Device price	Clinical items (converted into monetary units)	All costs (except device price)		Device price
	-positive impact	-negative impact	-negative impact	-positive impact	-negative impact		-negative impact
	(*) overall score^§^ for device A = A1 -A2 – A3			(*) overall score^§^ for device B = B1 - B2 -B3			
	Panel B: Two-scale design						
	Device A			Device B			
Qualitative analysis	Scale 0-70		Scale 0-30	Scale 0-70		Scale 0-30	
	Clinical items (converted into monetary units): parameter A1 (**)	All costs (except device price): parameter A2 (**)	Device price parameter A3 (**)	Clinical items (converted into monetary units): parameter B1 (**)	All costs (except device price): parameter B2 (**)	Device price parameter B3 (**)	
	-positive impact	-negative impact	-negative impact	-positive impact	-negative impact	-negative impact	
From qualitative to quantitative analysis	(**) result 0-70 for A (*)		(**) result 0-30 for A (*)	(**) result 0-70 for B (*)		(**) result 0-30 for B (*)	
	(*) result 0-100 for device A			(*) result 0-100 for device B			
Quantitative analysis	Clinical items (converted into monetary units)	All costs (except device price)	Device price	Clinical items (converted into monetary units)	All costs (except device price)	Device price	
	-positive impact	-negative impact	-negative impact	-positive impact	-negative impact	-negative impact	
	overall (reparameterized^§§^) score for device A = A1 -A2 – A3			overall (reparameterized§^§§^) score for device B = B1 - B2 -B3			
